# A Safe and Compliant Noncontact Interactive Approach for Wheeled Walking Aid Robot

**DOI:** 10.1155/2022/3033920

**Published:** 2022-03-16

**Authors:** Donghui Zhao, Wei Wang, Moses Chukwuka Okonkwo, Zihao Yang, Junyou Yang, Houde Liu

**Affiliations:** ^1^School of Electrical Engineering, Shenyang University of Technology, Shenyang, China; ^2^Shenyang Institute of Automation, Chinese Academy of Sciences, Shenyang, China; ^3^Center for Artificial Intelligence and Robotics, Tsinghua University, Shenzhen, China

## Abstract

Aiming at promptly and accurately detecting falls and drag-to gaits induced by asynchronous human-robot movement speed during assisted walking, a noncontact interactive approach with generality, compliance and safety is proposed in this paper, and is applied to a wheeled walking aid robot. Firstly, the structure and the functions of the wheeled walking aid robot, including gait rehabilitation robot (GRR) and walking aid robot (WAR) are illustrated, and the characteristic futures of falls and the drag-to gait are shown by experiments. To obtain gait information, a multichannel proximity sensor array is developed, and a two-dimensional gait information detection system is established by combining four proximity sensors groups which are installed in the robot chassis. Additionally, a node-iterative fuzzy Petri net algorithm for abnormal gait recognition is proposed by generating the network trigger mechanism using the fuzzy membership function. It integrates the walking intention direction vector by taking gait deviation, frequency, and torso angle as input parameters of the system. Finally, to improve the compliance of the robot during human-robot interaction, a PID_SC controller is designed by integrating the gait speed compensation, which enables the WAR to track human gait closely. Abnormal gait recognition and assisted walking experiments are carried out respectively. Experimental results show that the proposed algorithm can accurately identify abnormal gaits of different groups of users with different walking habits, and the recognition rate of abnormal gait reaches 91.2%. Results also show that the developed method can guarantee safety in human robot interaction because of user gate follow-up accuracy and compliant movements. The noncontact interactive approach can be applied to robots with similar structure for usage in walking assistance and gait rehabilitation.

## 1. Introduction

Due to an increase in the percentage of the aging population and the growing number of disabled people with lower limb impairment, there is a significant rise in the demand for walking aids or professional care attendants in the daily lives of the affected people. But this increasing demand cannot be sufficiently satisfied due to current shortage in supply [1, 2]. Hence, the development of a robot which can help in walking for both the elderly and disabled during rehabilitation has become a prominent issue in the field of robotics [3].

The compliant control of robots for assisted walking is an important research subject regarding the medical care needed for weak motion capability user groups. In recent years, researcher globally, have studied this and proposed several methods [4]. JR et al. proposed a robot walking control method based on COR (center of rotation), which enables physically impaired users to compliantly walk by changing the kinematic structure of the system [5]. Jamwal et al. proposed the impedance control method. This control method has small computation and strong robustness, and has been widely used in the compliant control field of WAR. However, when there is external disturbance in the human-robot interaction environment, this control system cannot maintain optimal impedance control throughout the whole process [6]. On this basis, Jiang et al. proposed a shared control method that takes the robot system and human-robot interaction as inputs, which can assign a degree of control to the user according to different use scenarios [7] (this control method change the main control source depending on the situation to either the user or the robot). Xu et al. proposed a shared control method based on reinforcement learning algorithm, which enables the robot to adapt to the user's personal characteristics and gait, thus making walking assistance more stable [8]. To improve the safety of the user during rehabilitation, Xu et al. proposed a compliant control method based on multisensor fusion technology. This method improved the compliance of the rehabilitation robot, nevertheless, the method has a poor walking intention recognition performance in an environment containing non-Gaussian noise [9]. Han et al. proposed trajectory tracking control method based on PID neural network to enhance the compliance in human-robot interaction [10]. Yan et al. developed a force sensor array to measure human-robot interaction force of the user's upper limbs to calculate required motion intention information. Laser sensors are then used to measure leg distance to predict the walking intention of the user's lower limbs. For compliant robot movements, the above obtained data are fused with Kalman filter algorithm to acquire the user's walking intention speed [11]. Hirata et al. proposed updating the state estimation parameters of the user to realize robot compliant motion control by observing the human-robot physical interaction which supports the user's assisted walking [12]. Song et al. developed an external force observer based on the measurement of motor current and speed. The robot adapts by moving compliantly according to the force applied by the user, but the robot cannot quickly process any instantaneous data like pulls and push caused by the user's fall or other emergency situations, thus increasing the risk of secondary injury during a fall [13, 14].

Although a large number of studies have been carried out on the compliant control of walking rehabilitation robots, extreme situations such as robot induced falls and drag-to gaits which have not been considered in the recognition of abnormal human walking intentions, makes it impossible to effectively guarantee absolute safety in human-robot interaction. Regardless of using robots as a walking aid or in more complex rehabilitation scenarios, ensuring user safety is an important factor to consider during research [15, 16]. If the robot has the ability to detect a fall before the patient reaches the ground thereby enabling its prevention, user safety will be ensured.

Aiming at this kind of safety issue, researchers have proposed some fall detection methods, such as wearable sensor detection [17, 18], visual detection [19, 20], and environmental monitoring [21, 22]. A wrist watch with built-in accelerometer can be used for fall detection by monitoring the amplitude of acceleration with matching support vector machine algorithm [17]. The fall detection algorithm based on the fusion of plantar pressure signal and surface EMG signal achieves an average recognition rate of 91.7% in normal day usage [23]. Huang et al. [24], Ma et al. [25], and Qiu et al. [18] put forth the body posture estimation algorithm based on wearable sensor which can effectively estimate the user's posture, detect abnormal behavior in real time and monitor the user online. Unfortunately, for the wearable fall recognition methods, special sensors need to be worn in advance to collect and store walking and fall data incidences which increases usage complexity and poor user experience. Consequently, Lee et al. proposed a method to estimate the user's walking state based on visual inspection [19]. Kalman recursive prediction based on real-time measurements of the knee angle actualizes effective abnormal gait monitoring [26]. This visual inspection method has both low cost and ease of use advantages as there is no need to wear any equipment. But the disadvantage is that it can only be used in the environment where sensors are installed. Li et al. proposed a method based on modified zero moment point for fall predictions and used Kinect sensor to monitor user's movements [21]. Di et al. proposed the estimation of the user's center of gravity in real time based on COP-FD algorithm and ZMP (zero moment point) algorithm [27, 28], so as to monitor the user's state online. Yan et al. proposed a human-robot cooperative stability algorithm to measure the walking state of both the robot and user [29]. Wakita et al. have designed a robot with a smart cane to help the elderly and disabled during walking. The concept of “intention direction” was proposed and various sensors are used to detect the user's intention. But the human-robot interface which uses multiaxis sensors is expensive and fragile, and not affordable for a large number of users [30]. In the process of using a wheeled walking aid robot, a neuromuscular disorder in the user's lower limb may lead to walking ability degeneration, abnormal gait, and body imbalance. Developments such as robot induced drag may be observed due to the failure of the patient to follow the robot in time. This generally led to falls causing dangerous secondary injuries. At present, there is no research on these particular robots induced abnormal gait which usually occurs before users fall. Meanwhile, the noncontact interaction mode is more convenient for the users with weak motion capability, which could avoid cumbersome steps, such as repeated wearing and data correction in advance [31, 32]. In particular, for gait rehabilitation training, the noncontact interaction mode enables users to actively master the gait rhythm and gait phase. It helps to promote the active participation of users and improve the effect of gait training [33].

To recognize abnormal gaits accurately, we proposed a node-iteration fuzzy petri net algorithm (NIFPN), which is applied in the gait rehabilitation robot (GRR) and walking aid robot (WAR). Additionally, we developed a compliant PID_SC controller, which can track the user's gaits accurately. On the whole, a noncontact interactive approach which ensures both safety and compliant motion is proposed. The method proposed in this paper has the following innovations:

1. A low-cost multichannel proximity sensor is developed to effectively detect real-time gait information by multichannel data fusion. Its unique noncontact design has good generalization characteristics, which means the sensor can be applied to wheeled walking aid robot with similar structure.
2. A node-iteration fuzzy petri net (NIFPN) algorithm is proposed to recognize abnormal gait. The recognition rate is improved by updating nodes for individual behavior differences of users. Compared with the traditional experience-based threshold method, this eliminates involved data precorrection and storage.
3. A PID_SC controller is proposed to adequately follow the user's gaits. Human gait speed compensation is introduced in the calculation process of traditional PID controller, which significantly improves the stability and compliance of WAR during assisted walking.

The rest of this paper is as follows: Section 2 analyzes the composition structure and functions of the developed WAR and discussed subsequent preliminary experiment processes in details.  Section 3 introduces the abnormal gait recognition data extraction method and proposes the NIFPN algorithm and PID_SC controller.  Section 4 and 5, respectively, describes the comprehensive experiment and discusses related conclusion.

## 2. Materials

**Figure 1:**
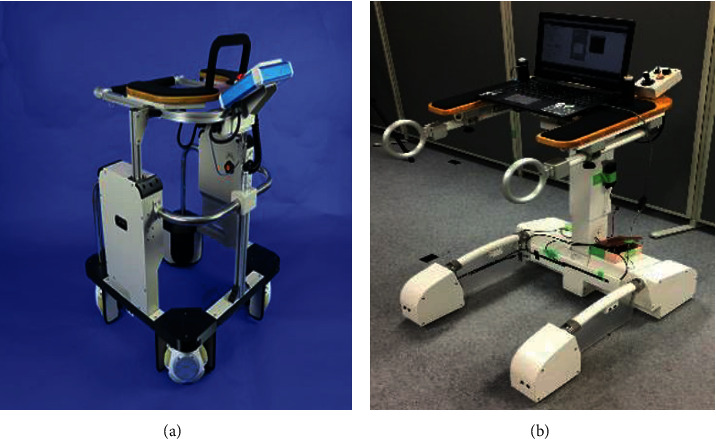
Wheeled walking aid robots. (a) GRR. (b) WAR.

Our laboratory has developed two wheeled walking aid robots: gait rehabilitation robot (GRR) and walking aid robot (WAR), as shown in Figure 1. WAR is specially made for assisting the elderly and patients with walking inabilities, while GRR mainly serves medial gait rehabilitation purposes. WAR will be used for the experimental demonstration of the above stated method in this paper.

### 2.1. Walking Aid Robot

**Figure 2:**
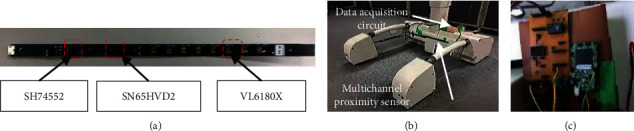
Gait information detection platform. (a) Multichannel proximity sensor. (b) Sensor installation. (c) Data acquisition circuit.

WAR is composed of an operation interface, pressure support plate and gait information detection platform. Four pressure sensors are embedded in the pressure support plate to obtain the direction intention [34]. To further improve the accuracy of information acquisition, we established a gait information detection platform using the multichannel proximity sensor, as shown in Figure 2. The multichannel proximity sensor developed is shown in Figure 2(a), which mainly consists of the laser distance measurement sensor VL6180X, embedded microcontroller SH74552, and CAN transceiver SN65HVD230. In placing two sensors in the front side of the gait information detection platform, we measure the distance between the legs as they swing back and forth. Two other sensors are installed on both sides of the platform to measure the lateral distances of both legs, as shown in Figure 2(b). Distance data from the multichannel proximity sensor is transmitted to the data acquisition circuit, as shown in Figure 2(c), where they are integrated and sent to a microcomputer. Received data are converted form CAN communication protocol to serial communication protocol by the MCU before being sent to the PC. The PC controls the movement of WAR based on the movement status of both legs.

### 2.2. Preliminary Preparation Experiment

**Figure 3:**
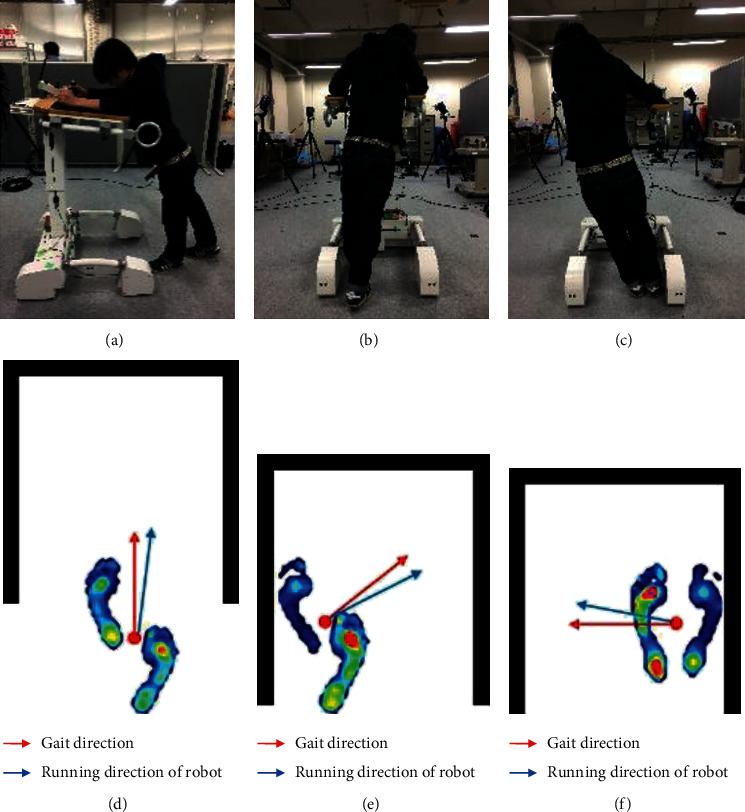
Experiment of multimodal assisted walk. (a) Front. (b) Right-front. (c) Left-front. (d) Forward gait. (e) Right front gait. (f) Left front gait.

To effectively detect the abnormal gait characteristics of people with lower limb disabilities. Multiple directional walking experiments and long-distance linear walking experiments were conducted respectively, as shown in Figure 3. More intuitive insight of the user's gaits is gotten by installing a pressure sensor array in the user's shoes, which effectively reflects the center of gravity and gait swing phase of the user's foot. To illustrate the directional interaction between user and robot, we take the forward movement of the user as the front cardinal direction with respect to the robot. Front, right-front and left-front walking directions are shown in Figures 3(a), 3(b), and 3(c), respectively, and the corresponding gait information are shown in Figures 3(d), 3(e), and 3(d). In the right-front walking direction as shown in Figures 3(b) and 3(e), the user's body pressure is concentrated on his right foot, which means that the person intends to initiate a forward right walking movement and to appropriately respond to this, WAR makes a right-front movement. At this time, if the user cannot swing the left foot quickly, a collision may occur or the robot will drag the user towards the corresponding direction. Also, in Figure 3(c), the center of gravity of the user is extremely inclined to the left leg, causing the right foot to easily collide with the side of the robot as the user is dragged along. Falls and induced drag-to gait may not only occur in the already discussed direction but also can occur and be detected as well in all directional movement of the user and robot. Through experiments, it can be observed that when the intention direction line of the user on the co-ordinate plane has a large degree of deviation with respect to the running direction of the robot, abnormal gaits will be induced if not adjusted in time. In practical usage, because of impairment in the lower limb muscles and nerves, or fatigue caused by long usage of the robot, it often happens that the user fails to keep up with the speed of the robot. Users with extreme conditions may fall to the ground because of their inability to self-adjust their gait speed.

## 3. Method

### 3.1. System Block Diagram

**Figure 4:**
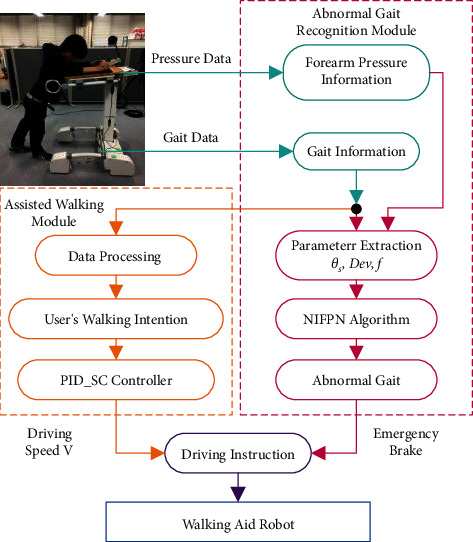
The system block diagram.

The WAR provides two operation modules, namely, assisted walking module and abnormal gait recognition module. As shown in Figure 4, an active compliant control method is introduced according to the following: first, the multichannel proximity sensor and pressure sensor on the robot collects the user's gait information and the forearm pressure information respectively. In assisted walking module, the gait information is passed through the data processing to complete the recognition of the user's walking intention. Information from the center position of the user's body is inputted to the PID_SC controller which outputs the driving speed used for the control of WAR during assisted walking.

For the abnormal gait recognition module, information obtained from the multichannel proximity sensor and pressure sensor which includes inclination angle, walking intention deviation angle and frequency serve as the input parameters of NIFPN algorithm. After these parameters are inputted to the algorithm, involved calculations and gait evaluations are done. The algorithm involves node updates which are essential for the optimization of abnormal gait recognition for individual users. When an abnormal gait is detected, the robot immediately brakes to prevent dragging the user. Finally, the driving speed output from the PID_SC controller is combined with the emergency braking indicator signal from abnormal gait recognition results to control the movement of WAR

### 3.2. Walking Intention Recognition and Parameter Extraction

When interacting with robots, walking direction intention and gait information directly reflects movement state.

**Figure 5:**
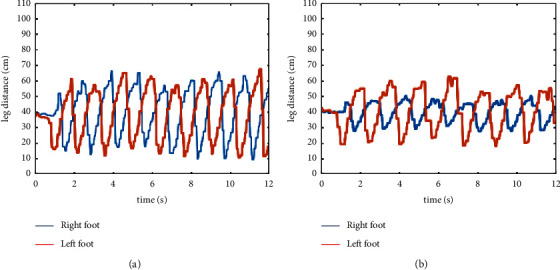
Gait information. (a) Normal walking. (b) Restrained walking.

**Figure 6:**
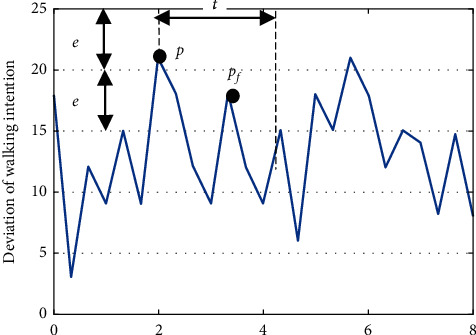
The definition of frequency parameter *f*.

(1) Gait information recognition: firstly, error data exceeding maximum distance of multichannel proximity sensor are excluded during the whole calculation. The mean value of the effective data obtain from the multichannel proximity sensor is taken as the observation value. Then, the relative distance between the foot contour and the robot is estimated based on the Kalman filter. The communication frequency of the whole system is 10 Hz.  Figure 5 shows the proximity data of the left and right feet during normal and restrained (user suffers an impairment on the left foot) walking. The vertical axis shows the distance measured by the proximity sensor, and the horizontal axis reflects time. For restrained walking (sensor data shown in Figure 5(a)), the blue signal line shows the position of the right foot with respect to the robot which is considered to support the body weight of the user because it has a shorter displacement. The red signal indicates a larger displacement of the left foot with respect to the robot. In this experiment, if the left foot is slow the user might not be able to follow or keep up with the speed of the robot.
Next, we propose a method to extract abnormal gait parameters and a coordinate system is established with its origin at the ground level of the left front corner of the base of the robot. The height of the robot given as *h*_*s*_.The coordinate of the force point of the combined upper limb pressure on the pressure support plate is *P*_*d*_(*x*_*d*_, *y*_*d*_, *h*_*s*_), and the combined vector *U* directly reflects the inclination degree of the torso, and thus serves as a safety indicator during assisted walking [35]. By selecting the upper left corner pressure sensor as the point of origin and the four sensor values as *p*_*fl*, *p*_*fr*, *p*_*bl*, and *p*_*br*, the walking intention direction vector force *F*(*f*_*d*_*,θ*) is obtain based on the distance type fuzzy inference algorithm [34]. The Euclidean distance between point *P*_*d*_(*x*_*d*_, *y*_*d*_) and the center point *P*_*s*_(*x*_*s*_, *y*_*s*_) of both feet reflects the deviation degree between the running direction of the robot and the actual footstep position. Again, in the case of abnormal gait, the user's lower limb cannot properly indicate the user's directional intentions which results in large driving angle and linear distance deviation. Three input parameters of the abnormal gait recognition system are as follows:
(2) Torso inclination angle *θ*_*s*_:
  (1) $$\begin{array}{c} \theta_{s}=\arctan\frac{\sqrt{{\left(x_{d}-x_{s}\right)}^{2}+{\left(y_{d}-y_{s}\right)}^{2}}}{h_{s}}. \end{array}$$
(3) Walking intention deviation parameter dev: the rate of change of the supporting force *f*_*d*_ of the arms on the pressure plates is *f*_*d*_′. When the pressure plates are supported with both arms, the rate of change of the supporting force *f*_*d*_′ will alternate irregularly with the intensity of movement. When the user is walking slowly, the center point *P*_*s*_ of both feet will fluctuate in a small range near *P*_*d*_.With increase in walking speed, the linear distance will also increase, and when a fall occurs, the linear distance will rise sharply. This value is taken as the gain of the rate of change of the supporting force, so as to effectively enlarge the deviation parameter of walking intention.
  (2) $$\begin{array}{c} \text{Dev}=f_{d}^{\prime }\cdot \sqrt{\left(x_{d}^{2}-x_{s}^{2}\right)+\left(y_{d}^{2}-y_{s}^{2}\right)}. \end{array}$$
(4) The fluctuation frequency *f* of walking intention deviation parameters: this is the sum of all frequencies of intention deviation within a certain range *e* in a period of time *t*. As shown in Figure 6. If the frequency is within this range, it means that the position of both feet still has a large deviation range from the intended direction. From this, we know that both legs did not swing in time and failed to follow the intention of walking direction.

According to the assisted walking experiment, the deviation between the extension line of the actual position and the direction intention is determined by the deviation amount Dev, and the inclination degree of the user's body is determined by the *z*-axis acceleration value. The frequency *f* reflects whether the user's gait can stably follow the intended direction in a given period of time.

### 3.3. Node-Iterative Fuzzy Petri Net Algorithm

Petri net has been widely used in fault diagnosis and other fields. It can effectively describe the dynamic process of abnormal phenomena and has the characteristic advantages of structured expression, quick inference, quick search and mathematical adaptation of diagnosis. The abnormal gait recognition process of a user while walking is a typical example of a dynamic process.

#### 3.3.1. Fuzzy Petri Net (FPN)

FPN is formed from the extension of the basic Petri net idea in [35, 36]. Each library of FPN is assigned a real value on [0,1] as its identification value and each transition is given a definite factor to represent the probability of transition occurrence. The input and output functions are also specified. Here FPN is defined with nine tuples:

(3) $$\begin{array}{c} \text{FPN}=\left\{P,T,D,I,O,\alpha ,\beta ,Th,U\right\}. \end{array}$$

Here, *P*={*p*_1_, *p*_2_,…, *p*_*m*_} is a finite set of repository nodes; *T*={*t*_1_, *t*_2_,…, *t*_*m*_} is a finite set of transition nodes; *D*={*d*_1_, *d*_2_,…, *d*_*m*_} is a finite set of propositions, and |*P*|=|*D*|, *P*∩*T*∩*D*=*ϕ*; *I*:*P*⟶*T* is the input matrix; reflects the mapping from library to transition. *I*={*δ*_*ij*_}, *δ*_*ij*_ is a logical quantity, *δ*_*ij*_ ∈ {0,1}, When *P*_*i*_ is the input of *T*_*j*_ (that is, there is a directional arc from *P*_*i*_ to *T*_*j*_), *δ*_*ij*_=1; otherwise *δ*_*ij*_=0, where *i*=1,2 … *n*, *j*=1,2 … *m*; *O* : *T*⟶*P*, is an output matrix, *O*={*γ*_*ij*_}, *γ*_*ij*_ is a logical quantity, *γ*_*ij*_ ∈ {0,1}, when *P*_*i*_ is the output of *T*_*j*_ (that is, there is a directional arc from *P*_*i*_ to *T*_*j*_), *γ*_*ij*_=1; otherwise *γ*_*ij*_=0, where *i*=1,2 … *n*,  *j*=1,2 … *m*; *α* : *P*⟶[0,1], indicates the confidence of the proposition corresponding to the library; *β* : *P*⟶*D*, is a mapping; reflects the corresponding relationship between the nodes of the library and the proposition; If *α*(*p*_*i*_)=*y*_*i*_, *y*_*i*_ ∈ [0,1], and *β*(*p*_*i*_)=*d*_*i*_, the confidence of proposition *d*_*i*_ is *y*_*i*_. *Th*: *Th*⟶[0,1], defines the domain value *λ*_*i*_ for transition node *t*_*i*_(*t*_*i*_ ∈ *T*), *Th*={*λ*_1_, *λ*_2_,…*λ*_*m*_}. *U*: rule confidence (CF) matrix, *U*=diag(*μ*_1_,…*μ*_*m*_), *μ*_*j*_ is the confidence of rule *T*_*j*_, *μ*_*j*_ ∈ [0,1].

**Figure 7:**
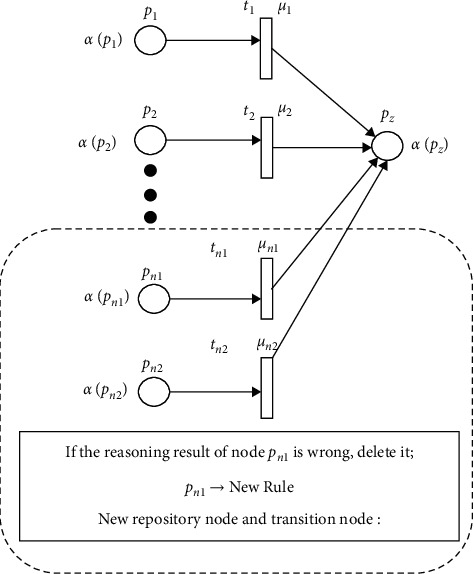
The NIFPN model.

FPN is a rule-based system, and its rules can be expressed with the corresponding FPN models. In the reasoning of abnormal gait during detection, the rules follow a MISO (multiple-input-single-output) FPN model, as shown in the formula: *R*_*i*_ :  IF *p*_1_ OR *p*_2_OR ⋯ OR *p*_*m*_; THEN *p*_*z*_(CF=*μ*_1_, *μ*_2_ … *μ*_*m*_), because of the possible individual differences between the fuzzy base rule system established for a particular user group and a single patient with mobility difficulties, reasoning accuracy of our algorithm could be reduced. Therefore, it is necessary to modify the base nodes and transition nodes in the original FPN model with nodes that are individual user centered. Please refer to next section for details of the specific node update methods. The corresponding node iterative FPN model is shown in Figure 7.

Among them, the confidence of proposition *p*_1_, *p*_2_ … *p*_*n*_ is *α*(*p*_1_), *α*(*p*_2_) … *α*(*p*_*n*_). In the fuzzy reasoning method, the fuzzy product rule is adopted, which describes the fuzzy relationship between the antecedent and result. *R* is a fuzzy set rule base, *R*={*R*_1_, *R*_2_,…*R*_*n*_}, *i* order of the fuzzy rule is *R*_*i*_.

#### 3.3.2. Reasoning Flow of the NIFPN

The system input Mem(*p*_*i*_)∀ *p*_*i*_ ∈ IP, *IP* is set by the input library while the system output is Mem(*p*_*i*_)∀ *p*_*i*_ ∈ OP, and OP is set by the output library. Calculation steps involved in node iterative FPN reasoning of abnormal gait are as follows:

**Step 1 .**

Initialization: fuzzy set is defined by membership degree where the initial labeling function is

(4) $$\begin{array}{c} M\left(p_{i}\right)=0,\text{ if }p_{i}\notin \text{IP,}\\ M\left(p_{i}\right)=\text{the number of data tokens, if }p_{i}\in \text{IP.} \end{array}$$

**Step 2 .**

Calculate the fuzzy relation matrix, i.e., ∀*t*_*j*_ ∈ *T*, *V*(*t*_*j*_)=*W*_*a*_ × *W*_*c*_=(*w*_*a*1_, *w*_*a*2_,…*w*_*am*_)^*T*^∧(*w*_*c*1_, *w*_*c*2_,…*w*_*cm*_), *V*(*t*_*j*_) is the fuzzy relation matrix between antecedent and result in a given time *t*_*j*_, *W*_*a*_={*w*_*a*1_, *w*_*a*2_,…*w*_am_} is the weight fuzzy set of the antecedent while *W*_*c*_={*w*_*c*1_, *w*_*c*2_,…*w*_*cm*_} is the weight fuzzy set of the result. Each element in a fuzzy set is represented by a fuzzy weight interval.

**Step 3 .**

Input the data for detection *W*_*a*−input_.

**Step 4 .**

Initiate transition, i.e., calculate

(5) $$\begin{array}{c} t_{j}\in \frac{T}{\forall p_{k}},\\ W_{a}^{\prime }=W_{a-\text{input}},\\ W_{c}^{\prime }=W_{a}^{\prime }*\circ V\left(t_{j}\right)\text{ or }\neg W_{a}^{\prime }\circ V\left(t_{j}\right). \end{array}$$

**Step 5 .**

Output: for the output variable *O*, its associated membership function is *W*_*c*_′={*w*_*ci*_′}=∨*w*_*c*_′, *i*=1,2,…*I*; *W*_*c*_′ is the system's output value.

**Step 6 .**

When the transition initiation conditions are met, return to Step 4, i.e., meet the following requirements:

(6) $$\begin{array}{c} \exists t_{j}\in \frac{T}{M\left(p_{i}\right)}=1,\;\forall p_{j}\in I\left(t_{j}\right). \end{array}$$

**Step 7 .**

Calculate the real operation value by using the maximum defuzzification method.

**Step 8 .**

If the reasoning result is wrong, delete the original node and regenerate it based on collected data and current state.

### 3.4. PID_SC Controller

**Figure 8:**
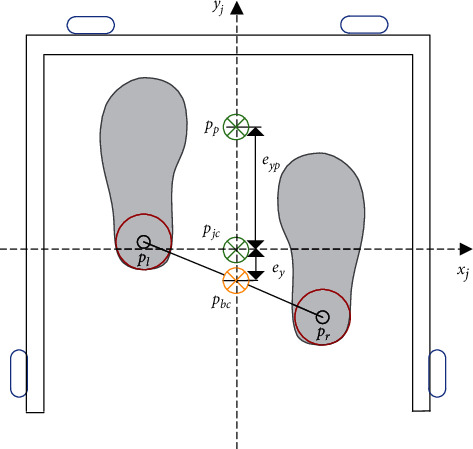
Motion information detection.

Using the direct distance controller results to a rough, unstable and unsafe movement in the robot [37]. This is caused by an intermittent or discrete motion of the robot in the *v*_SC_ and *y* relative position axes. The PID_SC controller proposed serves the purpose of making the motion of robot controller more compliant. As shown in Figure 8, two-dimensional cartesian coordinate system is constructed based on mutually perpendicular multichannel proximity sensors to improve the human-robot interaction process. The geometric center of the robot is *P*_*jc*_(*x*_*c*_, *y*_*c*_), the coordinates of the user's left tibia is *P*_*l*_(*x*_*l*_, *y*_*l*_), right tibia is *P*_*r*_(*x*_*r*_, *y*_*r*_), *P*_*p*_ is the next gait position. According to the line from point *P*_*l*_ and *P*_*r*_, and its midpoint *P*_*bc*_, we defined the body's center of gravity as *P*_*bc*_(*x*_*b*_, *y*_*b*_). During assisted walking, we expect *P*_*jc*_ and *P*_*bc*_ to stay overlapped with each other, that is, the center of the user's body is always near the geometric center of the robot, so as to avoid collision, drag-to gait or overall torso tilt.

First, the PID controller enables the robot to calculate the difference between *P*_*jc*_ and *P*_*bc*_. In the process of moving forward or backward, the position error with respect to *P*_*bc*_(*x*_*b*_, *y*_*b*_) is represented as *e*_*x*_, *e*_*y*_, and *e*_*yp*_ (with directions *x*_*j*_ and *y*_*j*_ as reference). They are defined as *e*_*x*_=*x*_*j*_ − *x*_*b*_ and *e*_*y*_=*y*_*j*_ − *y*_*b*_ respectively. In order to minimize the error, the controller is designed as

(7) $$\begin{array}{c} \left\{\begin{array}{c} {\dot{x}}_{b}=k_{P,x}e_{x}+k_{I,x}\int e_{x}\text{d}t+k_{D,x}{\dot{e}}_{x},\\ {\dot{y}}_{b}=k_{P,y}e_{y}+k_{I,y}\int e_{y}\text{d}t+k_{D,y}{\dot{e}}_{y}. \end{array}\right. \end{array}$$

where ${\dot{x}}_{b}$ and ${\dot{y}}_{b}$ are the input velocity of the system and *k*_*P*_, *k*_*I*_, and *k*_*d*_ represent the proportional gain, integral gain and differential gain, respectively. Although the PID is adopted for movement control in this paper, it is yet necessary to input the gait speed of the user to ensure compliance in motion. Reasons being that relative position error changes continuously due to the influence of continuous and unequal gait of the user. That is, during human-robot interaction, geometric relative positions are directly affected by all intermittent motions. Hence, we propose the addition of gait speed compensation in the originally established controller.

Gate speed compensation is mathematically expressed as follows: the user moves the right leg from the initial position to the next position at a distance of *d*_*sl*_ and time *t*, throughout which the left leg is fixed. The rule of thumb is that the step is about twice the displacement of *P*_*bc*_, that is, the absolute velocity *v*_*a*_=*d*_*sl*_/1.5*t*. The estimated position is *P*_*p*_(*x*_pc_, *y*_pc_), and then the error in the *y* direction is *e*_*yp*_+*e*_*y*_. In the right leg movement, the estimated speed *v*_*p*_ is

(8) $$\begin{array}{c} v_{p}=\frac{e_{yp}+e_{y}}{t}. \end{array}$$

Gait speed compensation *v*_SC_ is calculated by combining the estimated gait speed *v*_*p*_ and the absolute speed *v*_*a*_ expressed as

(9) $$\begin{array}{c} v_{SC}=\frac{d_{sl}+3e_{yp}+3e_{y}}{6t}. \end{array}$$

For forward or backward movement, the PID_SC controller output is expressed as

(10) $$\begin{array}{c} {\dot{y}}_{b}=k_{P,y}e_{y}+k_{I,y}\int e_{y}\text{d}t+k_{D,y}{\dot{e}}_{y}+v_{SC}. \end{array}$$

## 4. Experiment and Analysis

The effectiveness of the proposed method is investigated by carrying out comprehensive experiments. 30 subjects with limited mobility (15 males and 15 females) were invited to carry out abnormal gait recognition experiments and assisted walking experiment. Then, the proposed method was tested in smart house to evaluate the safety and comfort of users. Finally, a comparative analysis was carried out to prove the superiority of the proposed method.

### 4.1. Abnormal Gait FPN Model

**Figure 9:**
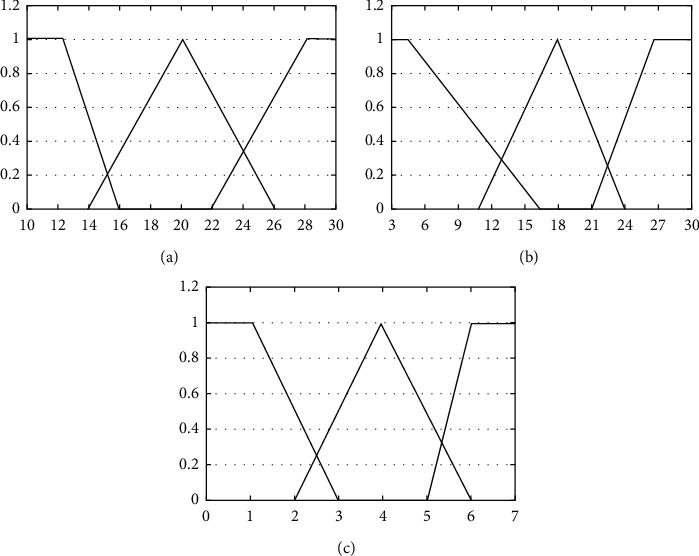
Membership function for input parameter. (a) Membership function for Dev. (b) Membership function for *z*. (c) Membership function for *f*.

According to the walking intention recognition and parameter extraction, the membership function is defined by the deviation Dev, *z*-axis acceleration, and frequency *f*. The function is divided into three states: high, medium, and low. To meet actual requirements high, medium, and low membership function parameters are divided according to the average deviation, as shown in Table 1, and their corresponding membership functions are shown in Figure 9.

In this paper, the logical relationships among the gait information, position and movement direction deviations, and parameters with self-adjustment capabilities are simplified based on FPN and represented with “library” and “transition” nodes which are connected by directional arcs. Experimental analysis shows that the value of Dev is usually between 8 and 30, the body inclination angle *z* decreases to values between 3 and 28, and the frequency *f* is between 1 and 10. When walking occurs slowly, Dev is usually between 10 and 16, inclination angle *z*, between 5 and 16, and frequency *f*, and between 5 and 6. During a fall, *De*  *v* usually has values of range 22 ∼ 28, the torso inclination angle *z* will be between 21 and 27 and the frequency *f* decrease to a range of 1 ∼ 3. Because the member function is required to be between 0 and 1, the three input parameters are normalized to the range of 0 to 1. *α*, *β*, and *γ* represent Dev, *z*-axis acceleration and frequency parameter *f*, respectively. *H*, *M*, and *L* represent the membership functions of “high,” “medium,” and “low.”

The fuzzification process is defined as follows: three fuzzy rules are formulated to correspond with three fuzzy results: normal, fast and abnormal walking gait. Next, we setup and configure input language variables, deviation Dev, torso inclination angle *z* and frequency parameter *f*. The different language variables are defined as high, medium and low. Fuzzy rules are

(11) $$\begin{array}{c} R_{1}:\text{if Dev is }L\text{ and }z\text{ is }L\text{ and }f\text{ is }H\text{ Then }D\;i\text{s NA,}\\ R_{3}:\text{if Dev is }H\text{ and }z\text{ is }H\text{ and }f\text{ is }L\text{ Then }D\text{ is }F. \end{array}$$

**Figure 10:**
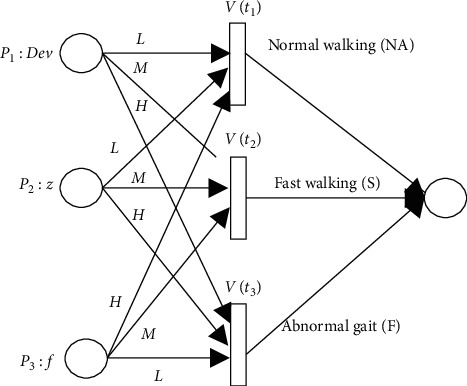
The fuzzy Petri model of abnormal gait detection.

FPN transformation result based on the above fuzzy rules is shown in Figure 10.

### 4.2. Case Analysis of Abnormal Gait

For illustrative purposes, we use the above mention FPN calculation steps to analyze abnormal gaits. The matrix of Dev, *z* and *F* parameters in the reasoning process is Dev, *z*, and *F*, respectively.

**Step 9 .**

Set the fuzzy set according to the experiment:

(12) $$\begin{array}{c} {\text{Dev}}_{L}=\frac{0.35}{{\text{dev}}_{ll}}+\frac{0}{{\text{dev}}_{lm}}+\frac{0}{{\text{dev}}_{lh}},\\ {\text{Z}}_{L}=\frac{0.27}{z_{ll}}+\frac{0}{z_{lm}}+\frac{0}{z_{lh}},\\ {\text{F}}_{M}=\frac{0.42}{f_{ml}}+\frac{0}{f_{mm}}+\frac{0}{f_{mh}},\\ {\text{Dev}}_{M}=\frac{0}{{\text{dev}}_{ml}}+\frac{0.58}{{\text{dev}}_{mm}}+\frac{0}{{\text{dev}}_{mh}},\\ Z_{M}=\frac{0}{z_{ml}}+\frac{0.49}{z_{mm}}+\frac{0}{z_{mh}},\\ F_{H}=\frac{0}{f_{hl}}+\frac{0.60}{f_{hm}}+\frac{0}{f_{hh}},\\ {\text{Dev}}_{H}=\frac{0}{{\text{dev}}_{hl}}+\frac{0}{{\text{dev}}_{hm}}+\frac{0.81}{{\text{dev}}_{hh}},\\ {\text{Z}}_{H}=\frac{0}{z_{hl}}+\frac{0}{z_{hm}}+\frac{0.71}{z_{hh}},\\ F_{L}=\frac{0}{f_{lh}}+\frac{0}{f_{lm}}+\frac{0.76}{f_{ll}},\\ \text{Status}=\frac{0.35}{s_{l}}+\frac{0.5}{s_{m}}+\frac{0.75}{s_{h}}. \end{array}$$

**Step 10 .**

Calculate the Cartesian product of the antecedent and the result to obtain the fuzzy relation matrix;

(13) $$\begin{array}{c} P_{1}={\text{Dev}}_{L}\times Z_{L}\times F_{M}={\left({\left(\begin{array}{ccc} 0.35 & 0 & 0 \end{array}\right)}^{T}\wedge \left(\begin{array}{ccc} 0.27 & 0 & 0 \end{array}\right)\right)}^{T}\wedge \left(\begin{array}{ccc} 0.42 & 0 & 0 \end{array}\right)\\ ={\left[\begin{array}{ccc} 0.27 & 0 & 0\\ 0 & 0 & 0\\ 0 & 0 & 0 \end{array}\right]}^{T}\wedge \left(\begin{array}{ccc} 0.42 & 0 & 0 \end{array}\right)=\left[\begin{array}{ccc} 0.27 & 0 & 0\\ 0 & 0 & 0\\ 0 & 0 & 0 \end{array}\right],\\ P_{2}={\text{Dev}}_{M}\times Z_{M}\times F_{H}={\left({\left(\begin{array}{ccc} 0 & 0.58 & 0 \end{array}\right)}^{T}\wedge \left(\begin{array}{ccc} 0 & 0.49 & 0 \end{array}\right)\right)}^{T}\wedge \left(\begin{array}{ccc} 0 & 0.6 & 0 \end{array}\right)\\ ={\left[\begin{array}{ccc} 0 & 0 & 0\\ 0 & 0.49 & 0\\ 0 & 0 & 0 \end{array}\right]}^{T}\wedge \left(\begin{array}{ccc} 0 & 0.6 & 0 \end{array}\right)=\left[\begin{array}{ccc} 0 & 0 & 0\\ 0 & 0.49 & 0\\ 0 & 0 & 0 \end{array}\right],\\ P_{3}={\text{Dev}}_{H}\times Z_{H}\times F_{L}={\left({\left(\begin{array}{ccc} 0 & 0 & 0.81 \end{array}\right)}^{T}\wedge \left(\begin{array}{ccc} 0 & 0 & 0.71 \end{array}\right)\right)}^{T}\wedge \left(\begin{array}{ccc} 0 & 0 & 0.76 \end{array}\right)\\ ={\left[\begin{array}{ccc} 0 & 0 & 0\\ 0 & 0 & 0\\ 0 & 0 & 0.71 \end{array}\right]}^{T}\wedge \left(\begin{array}{ccc} 0 & 0 & 0.76 \end{array}\right)=\left[\begin{array}{ccc} 0 & 0 & 0\\ 0 & 0 & 0\\ 0 & 0 & 0.71 \end{array}\right]. \end{array}$$

∀*t*_*j*_ ∈ *T*, *T* is the transition set. Calculate fuzzy relation matrix *V*(*t*_*j*_), which is the matrix of fuzzy antecedent and rule *t*_*j*_, as follows:

(14) $$\begin{array}{c} V\left(t_{1}\right)=\left[\begin{array}{ccc} 0.27 & 0 & 0\\ 0 & 0 & 0\\ 0 & 0 & 0 \end{array}\right]\in P_{1}\times \text{Status}\times s_{l},\\ V\left(t_{2}\right)=\left[\begin{array}{ccc} 0 & 0 & 0\\ 0 & 0.49 & 0\\ 0 & 0 & 0 \end{array}\right]\in P_{2}\times \text{Status}\times s_{m},\\ V\left(t_{3}\right)=\left[\begin{array}{ccc} 0 & 0 & 0\\ 0 & 0 & 0\\ 0 & 0 & 0.71 \end{array}\right]\in P_{3}\times \text{Status}\times s_{h}. \end{array}$$

**Step 11 .**

Enter the set to be detected

(15) $$\begin{array}{c} \text{Dev}\prime =\frac{0}{{\text{dev}}_{l}}+\frac{0.02}{{\text{dev}}_{m}}+\frac{0.65}{{\text{dev}}_{h}},\\ Z\prime =\frac{0}{z_{l}}+\frac{0}{z_{m}}+\frac{0.63}{z_{h}},\\ \text{F}\prime =\frac{0}{f_{m}}+\frac{0}{f_{h}}+\frac{1}{f_{l}}. \end{array}$$

**Step 12 .**

Transition trigger changes

(16) $$\begin{array}{c} S_{1}^{\prime }=\text{Dev}\prime \circ V\left(t_{1}\right)\circ Z^{\prime }\circ F^{\prime }=\left[\begin{array}{ccc} 0 & 0.02 & 0.65 \end{array}\right]\circ \left[\begin{array}{ccc} 0.27 & 0 & 0\\ 0 & 0 & 0\\ 0 & 0 & 0 \end{array}\right]\circ \left[\begin{array}{ccc} 0 & 0 & 0.63 \end{array}\right]\circ \left[\begin{array}{ccc} 0 & 0 & 1 \end{array}\right]=\left[\begin{array}{ccc} 0 & 0 & 0 \end{array}\right],\\ S_{2}^{\prime }=\text{Dev}\prime \circ V\left(t_{2}\right)\circ Z^{\prime }\circ F^{\prime }=\left[\begin{array}{ccc} 0 & 0.02 & 0.65 \end{array}\right]\circ \left[\begin{array}{ccc} 0 & 0 & 0\\ 0 & 0.49 & 0\\ 0 & 0 & 0 \end{array}\right]\circ \left[\begin{array}{ccc} 0 & 0 & 0.63 \end{array}\right]\circ \left[\begin{array}{ccc} 0 & 0 & 1 \end{array}\right]=\left[\begin{array}{ccc} 0 & 0.20 & 0 \end{array}\right],\\ S_{3}^{\prime }=\text{Dev}\prime \circ V\left(t_{3}\right)\circ Z^{\prime }\circ F^{\prime }=\left[\begin{array}{ccc} 0 & 0.02 & 0.65 \end{array}\right]\circ \left[\begin{array}{ccc} 0 & 0 & 0\\ 0 & 0 & 0\\ 0 & 0 & 0.71 \end{array}\right]\circ \left[\begin{array}{ccc} 0 & 0 & 0.63 \end{array}\right]\circ \left[\begin{array}{ccc} 0 & 0 & 1 \end{array}\right]=\left[\begin{array}{ccc} 0 & 0 & 0.63 \end{array}\right]. \end{array}$$

**Step 13 .**

The results of fuzzy reasoning are as follows:

(17) $$\begin{array}{c} D=S_{1}^{\prime }\cup S_{2}^{\prime }\cup S_{3}^{\prime }=\frac{0}{d_{1}}+\frac{0.02}{d_{2}}+\frac{0.63}{d_{3}}. \end{array}$$

**Step 14 .**

The actual selected value is determined by using the maximum defuzzification method. Because *d*_3_ has the maximum membership degree, the case is regarded as a fall.

**Step 15 .**

Collect the abnormal gait of a subject whose right knee is fixed when he or she falls. The system's input data set to be analyzed for detection is

(18) $$\begin{array}{c} {\text{Dev}}^{\prime }=\frac{0}{{\text{dev}}_{l}}+\frac{0.5}{{\text{dev}}_{m}}+\frac{0.17}{{\text{dev}}_{h}},\\ Z^{\prime }=\frac{0}{z_{l}}+\frac{0.17}{z_{m}}+\frac{0.33}{z_{h}},\\ F^{\prime }=\frac{0}{f_{m}}+\frac{0}{f_{h}}+\frac{1}{f_{l}}. \end{array}$$

According to the same reasoning method, the result of the system is

(19) $$\begin{array}{c} D=S_{1}^{\prime }\cup S_{2}^{\prime }\cup S_{3}^{\prime }=\frac{0}{d_{1}}+\frac{0.17}{d_{2}}+\frac{0.17}{d_{3}}. \end{array}$$

**Figure 11:**
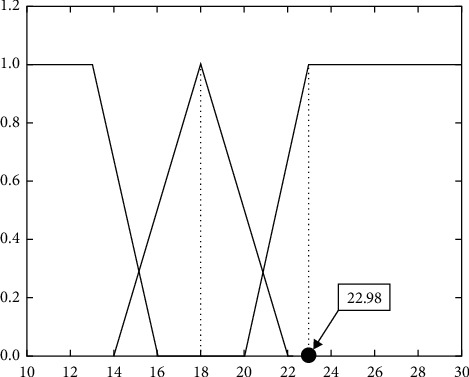
Membership function for input parameter.

That is to say, fall and fast walking inference probability obtained by the system is obviously inconsistent with the falling situations of the subject. The reason is that the Dev value of 22.98 of an abnormal gait is more similar to the fast gait of healthy subjects. Because the knee is limited and the gait state is different from that of healthy people, the inference accuracy will be reduced by using the same inference parameters. Therefore, it is necessary to adaptively update the transition trigger parameters in FPN. The updating method is shown in Figure 11. The membership degree of 22.98 in *de*  *v*_*h*_ is set as 1, and the membership degree of *de*  *v*_*m*_ in 22 is set as 0, so that the membership degree of *de*  *v*_*m*_ moves to the left as a whole with 18 as the center. That is, the membership degree corresponding to different values is replanned, thus updating the nodes confidence. The reasoning result after node update is

(20) $$\begin{array}{c} D=S_{1}^{\prime }\cup S_{2}^{\prime }\cup S_{3}^{\prime }=\frac{0}{d_{1}}+\frac{0.17}{d_{2}}+\frac{0.33}{d_{3}}. \end{array}$$

That is, the actual value determined by the maximum defuzzification method is of an actual fall situation.

### 4.3. Abnormal Gait Recognition Experiment

**Figure 12:**
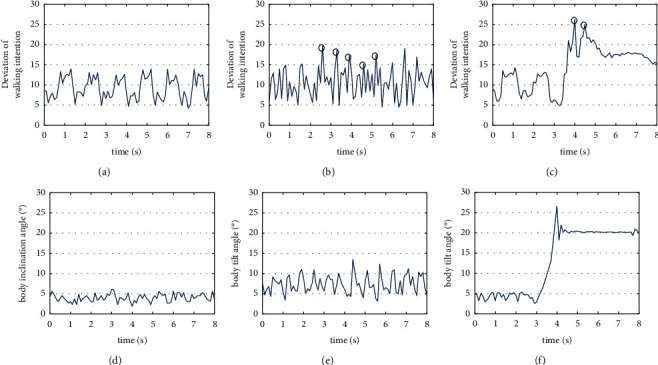
Normal walk, fast walk and fall of subject. (a) Dev of normal walk. (b) Dev of fast walk. (c) Dev of fall. (d) Z of normal walk. (e) Z of fast walk. (f) Z of fall.

Ten subjects wore devices at their knees to imitate the daily gait of patients with lower limb impairment to conducted indoor normal speed (0.27 m/s), fast speed (0.58 m/s), and abnormal gait walking experiments. The values *e*=4 and *t*=3 were constant.  Figure 12 shows the deviation parameters of walking intention of the subject when using the robot to perform experiment in the above listed conditions. When a subject is walking normally, the deviation parameters of the walking intention were consistent. As shown in Figures 12(b) and 12(e), the five peaks of Dev are 19.32, 18.79, 17.53, 15.26, and 17.88, which occur at 2.6 s, 3.2 s, 3.9 s, 4.6 s, and 5.2 s respectively. Therefore, the maximum value, 19.32, of Dev, and the maximum value, 13, of the torso inclination angle *Z* within 3 seconds period, both serves as the system input. Hence, the system inputs are Dev=19.32, *z*=13, and *F*=5, the reasoning results of the system are *S*_1_=0, *S*_2_=0.45, and *S*_3_=0. Final results obtained from maximum defuzzification method indicates that it was a fast speed walking case. Similarly, as shown in Figures 12(c) and 12(f), when a drag-to gait occurred, the two peaks of Dev are 26.91 and 25.01, which happen at time 3.9 s and 4.3 s, respectively. Again, the maximum value of Dev which is 26.91 and the maximum value of body torso inclination angle *Z* within 1 second period which is 27, both serve as the system's input. With Dev=26.91, *z*=27, and *F*=2, the reasoning result of the system is *S*_1_=0, *S*_2_=0.2, and *S*_3_=0.43. Final results obtain from maximum defuzzification method indicates that this is an abnormal gait walking case.

**Figure 13:**
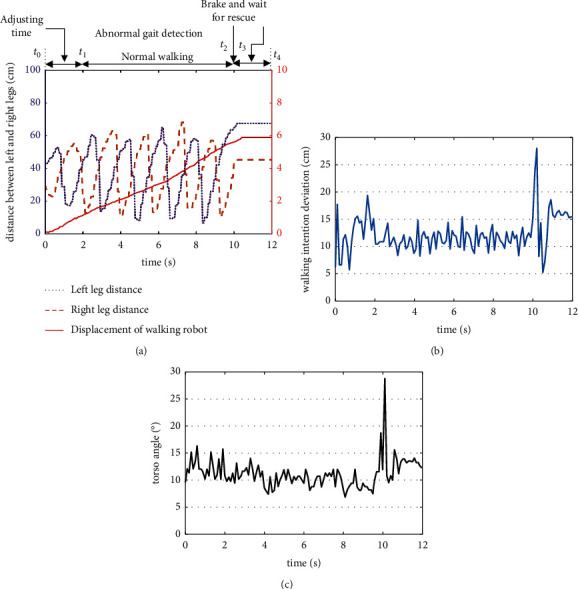
Parameter's variation of normal walk of health people. (a) Gait information and robot displacement. (b) Deviation parameter Dev of walking intention. (c) Slope angle *Z* of body.

Figure 13 shows the abnormal gait recognition experimental data result process of a subject with a fixed right knee. The experiment begins when the subject is at a standstill position. From *t*_0_ to *t*_1_, the distance between both legs changes abruptly. Also, the intention direction deviation degree changes greatly because the subject is constantly adjusting his gait to balance the upper body from the start to the end of the test process with the robot. Hence, data from the first 2 seconds of the experiment were not passed to the system as input until *t*_2_ when the subject begins to walk normally. The drag-to gait occurring within *t*_2_ to *t*_3_ was recognized by the system. Here, the peak value of Dev is 28.93, time at 10.1 s. So, the input of the system are Dev=28.03, *z*=29, and *F*=1. The system's reasoning are *S*_1_=0, *S*_2_=0, and *S*_3_=0.78. After maximum defuzzification, reasoning results indicate that it is an abnormal walking gait. Thus, at *t*_3_, the robot brakes urgently and rings a rescue alarm while the subject waits for rescue.

We comprehensively compare the NIFPN algorithm with SVM algorithm[38, 39]. Taking the falls and daily routines of general users into consideration, the abnormal gait test was conducted. To ensure the safety of the whole experiment, an elastic bandage is fixed between the subject and the robot to ensure that all the subject will not fall completely. The abnormal gait are categorized into forward falls, backward falls, vertical falls, and sideway falls, and drag-to gaits. We compare the two algorithms using the accuracy rate and misidentification rate. The conducted experiments and results are summarized in Table 2.

The accuracy percentage of abnormal gait of NIFPN is up to 91.2%, and the recognition rate of drag-to gait is much higher than SVM algorithm particularly. The misidentification percentage of daily routines is 6.27%. The majority of fall misjudgment is more likely to occur with sideway fall due to the relatively lower SVM generated at sideway fall, and therefore more easily to lead to misjudgment. Through the above experiments, the advantages of NIFPN algorithm are summarized as follows:

1. The input parameters of the algorithm reflect the relative position information between the user and the robot, and it replaces the experience-based threshold in traditional fall recognition methods. Therefore, this algorithm does not need to store the abnormal behavior gait data of users in advance, which improves the universality of the algorithm.
2. The algorithm introduces node iteration algorithm, which can effectively solve the differences of user with different gait habit. The recognition rate is improved by adaptive updating of nodes.
3. The algorithm can effectively recognize the drag-to gait, which is more suitable for the real use scenario.

### 4.4. Assisted Walking Experiment

**Figure 14:**
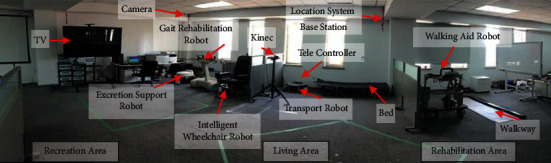
Smart house scenario based on multiply welfare robots.

To better serve the elderly and disabled, the laboratory setup a smart house environment [40] with a variety of welfare robots, such as gait rehabilitation robot, walking aid robot, intelligent wheelchair robot, transport robot, and excretion support robot as shown in Figure 14. It consists of 3 areas: recreation area, living area, and rehabilitation area. The control methods proposed in this paper are integrated in these robots.

To verify the effectiveness of the proposed noncontact compliant control method, 4 subjects conducted a multidirectional trajectory tracking experiment. The subjects walked in 8 directions assisted by the WAR according to preset trajectory. The walking path are made in square and diamond shapes with side length of 2 meters each. At the same time, some areas were marked with yellow markers, indicating that subjects should slow down when crossing these areas.

**Figure 15:**
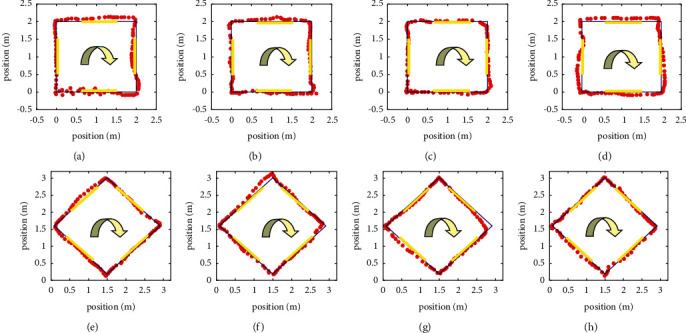
Preset path following experiment. (a) Square path of subject A. (b) Square path of subject B. (c) Square path of subject C. (d) Square path of subject D. (e) Diamond path of subject A. (f) Diamond path of subject B. (g) Diamond path of subject C. (h) Diamond path of subject D.

Figure 15 comparatively shows the path results of the four subjects while using WAR with respect to the target walking path. Although there are slight differences between the walking trajectory of the subjects and the preset trajectory, experimental results show that the method can accurately identify the subjects' walking intention direction and thus is able to satisfy rehabilitative needs.

**Figure 16:**
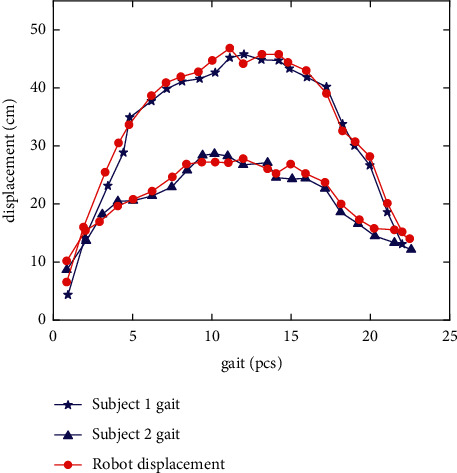
Experimental results of gait following.

Figure 16 shows the “slow-fast-slow” walking gait results of two subjects along the preset path. Although the subjects have individual differences, the robot can closely follow their walking gait.

**Figure 17:**
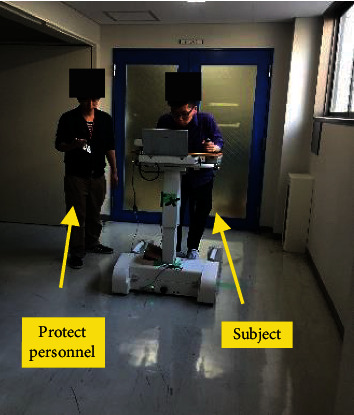
Auxiliary walking experiment.

To verify the superiority of the proposed method, the contrast experiments between the PID_SC controller, traditional PID controller and DDC (direct detection controller [37]) were implemented, as shown in Figure 17. Before the experiment, subjects were first allowed to use the robot for half an hour to ensure that they were fully familiar with the WAR operation mode. To ensure safety the maximum driving speed of WAR was set to 1.1 m/s. Then, all subjects were asked to walk a distance of 20 meters along the preset path using three compared control methods, respectively. The gait data and robot displacement data were recorded.

**Figure 18:**
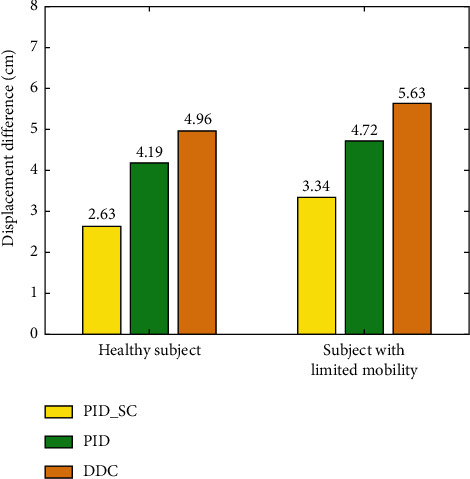
The contrast experiments of the PID_SC, PID, and DDC.

The comparative data of all the subjects with three control methods are shown in Figure 18. The displacement differences of all healthy subjects by PID_SC, PID, and DDC are 2.63 cm, 4.19 cm, and 4.96 cm, respectively. The displacement differences of subjects with limited mobility are 3.34 cm, 4.72 cm, and 5.63 cm, respectively.

The experimental results show that the displacement error of the compliant control method proposed in this paper is smaller than that of the traditional PID controller and direct distance controller. Faced with subjects with different motion capabilities, the proposed controller can control the WAR to produce movement closely corresponding to the user's walking gait. It is observed that the gait length and frequency of the healthy subjects can maintain a high consistency (almost uniform in speed), while the gait length and frequency of the subjects with limited mobility have more observable changes (long or short gait, and frequency fluctuation). Therefore, the overall displacement error of the subjects with limited mobility is slightly larger than that of the healthy subjects. Compared with the traditional control method, the controller introduces human gait speed compensation to reduce the relative displacement error between the robot and the user. Meanwhile, it also reduces the motion jam phenomenon and makes the motion process more flexible.

### 4.5. Comparison Analysis Experiment

To test the effectiveness of walking intention-based compliant control in gait rehabilitation, Tekscan Walkway footpath detection system was used to conduct assisted walking gait phase analysis experiment on test subjects [41]. We selected 20 subjects to participate in this experiment. All subjects were informed in advance and they agreed to all the test procedures of the experiment. In common practice gait phase is divided into eight. Since the subject's foot does not bear any pressure during the initial swing phase, the middle swing phase and the final swing phase while walking, these three phases were uniformly referred to as the swing phase for convenience in subsequent work.  Table 3 shows the data results of the subjects when performing compliant assisted walking compared to the passive assisted walking with the robot.

The data in the table are expressed in mean ± standard deviation. Perform difference analysis on the data in the table. ^*∗*^*P* < 0.05 indicates significant difference, and ^*∗∗*^*P* < 0.01 indicates extremely significant difference. For passive assisted walking, the movement path and speed of the robot are set in advance while the subject follows the robot to complete the rehabilitative walking exercise. The experimental results showed that the single leg support time and double leg support time of the subject were significantly increased when the compliant walking aid was used. Compared with passive assisted walking, the initial leg support time and swing time of the nonaffected leg increased significantly, while other time parameters showed no significant difference. The contact area, pressure and peak pressure of the two assistance methods are shown in Table 4.

The contact area between the midfoot and the full foot of the affected side increased significantly when the subject used robotic assisted compliant walking, but there was no significant difference between the forefoot and the rear foot. At the same time, there was a significant increase in the subject's midfoot and full foot contact pressure value. The nonaffected midfoot pressure and the peak pressure was higher in the compliant assisted walking than in the passive assisted walking through this index was not obvious on the affected side. These results indicated that subjects can actively walk and master their own gait rhythm during walking rehabilitation which significantly improves the symmetry and stability of the user's gait. Additionally, the improved safety protective measures in human-robot interaction provides psychological guarantee and reduces the mental and physical burden associated in using the robot for passive assisted walking. Overall, subjects show a boost in confidence while using GRR in walking which is necessary for medical rehabilitative recovery [42].

The asymmetrical analysis of all subjects' gaits is shown in Table 5. When subjects are assisted with robot compliance, the asymmetrical index of contact area, standing time, and swing time of both lower limbs were significantly improved compared to that of the passive assistance, given that the asymmetry index of contact pressure and track length has no significant difference. Results shows that robot-assisted gait training meets rehabilitation requirement and can significantly improve the user's gaits symmetry and stability.

Because the gait information detection system developed in this paper can be directly integrated into the mobile chassis and support plate, it can be directly applied to the wheeled walking aid robot with similar structure, and has good universality. The method proposed in this paper was compared with traditional identification methods which require special wearable motion detection device such as pressure sensor and gyroscope, as shown in Table 6.

**Figure 19:**
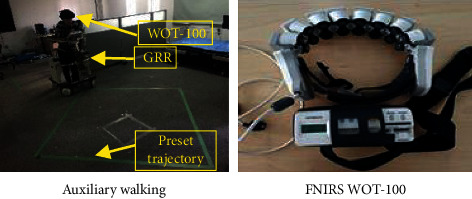
Acceptability experiment of human-robot interaction. (a) Auxiliary walking. (b) FNIRS WOT.

The approach used in this paper which is based on proximity sensor and pressure sensor basically has the same recognition rate as other abnormal gait recognition methods. Again, it is worth mentioning that this method can recognize all occurring drag-to gaits, and since this method does not require the user to wear any sensor, it increases the user's comfort and convenience. From the subjective point of view of users, this section makes a quantitative investigation on the comfort and acceptability of noncontact interaction methods. Aiming at measuring the robot-induced stress on humans during coexistence, subjective evaluation is usually acquired [43, 44]. During the comprehensive experiment, the comfortable feeling of different interaction methods is evaluated by a questionnaire result from all the subjects, which verifies the effectiveness and comfort of the proposed method. In the one-to-six scale, a higher score means a better comfortable feeling.  Table 7 shows the score change of comfortable feeling from the questionnaire survey. The “↑” represents an improvement in comfort, and the “=” and “↓” represent no significant change in comfort or less comfort than the previous methods. From this survey, we can find that most subjects felt more comfortable after an adjustment than traditional wearable methods before. This is because the proposed algorithm is based on the noncontact interaction method. The user can control the robot naturally without the repeated steps of placing the wearable sensor. Meanwhile, brain monitoring techniques have the capability to detect and characterize the operator's mental state such as workload, fatigue, or mental stress [45, 46]. It has been applied to assisted driving and assisted rehabilitation training for behavior correction and enhancing the acceptability of human-robot interaction. In this paper, Functional Near-InfraRed Spectroscopy (fNIRS) WOT-100, a brain imaging system to perform a continuous measure of the mental state, is introduced to monitor the user's mental fatigue when implementing noncontact interaction method and traditional method, as shown in Figure 19.

The mean and peak values of oxygenated signal are extracted as classification features, and the continuous autonomous assisted behaviors are classified combined with linear discriminant classifier LDA (linear discriminant analysis). As a method to evaluate mental fatigue, the classification results can directly distinguish the mental states of two different difficulty levels of behavior. The experiments of noncontact interaction method and traditional wearable interaction methods were carried out on 20 subjects for seven times. The classification results are shown in Table 7. The classification results of cerebral blood oxygen parameters of two tasks with different difficulty levels are large, and the difference is obvious. The greater physical exertion, weak action ability and balance ability subjectively cause the psychological load of subjects on risk behaviors such as falls, and increase the fluctuation of cerebral blood oxygen parameters. The noncontact interaction method can significantly reduce the physical consumption and mental fatigue of the subjects.

In general, the advantages of the noncontact interaction method proposed in this paper are summarized as follows:

1. The proposed method can ensure the user's direct operation, and avoids the cumbersome steps of repeated wearing and data correction. It enhances the user's comfort and convention.
2. For gait rehabilitation training, the proposed method helps users to walk actively and master their gait rhythm during rehabilitation process, which significantly improves the symmetry and stability of the user's gait.
3. The noncontact interaction method provides psychological guarantee, and reduces the mental and physical burden associated in using the robot. Overall, subjects show a boost in confidence while interacting with the robot with our proposed method, which is necessary for auxiliary walking and medical rehabilitative recovery.

## 5. Conclusion

This paper proposes a safe and compliant noncontact interactive approach for the wheeled walking aid robot. First, combined with the mechanical structure of wheeled walking aid robot, an expandable multichannel proximity sensor is designed. These sensors are combined and installed in the robot mobile chassis to recognize human gait information effectively. Secondly, a noncontact abnormal gait recognition approach based on NIFPN algorithm is proposed which identifies asynchronous human-robot movement speed or physical impairment induced falls and drag-to gaits during walking, enabling the robot to brake in emergency situations so as to ensure the safety of the user. Then, a PID_SC controller which integrates gait speed compensation feature is designed to accurately and compliantly follow the user's gaits. Experimental results show that the NIFPN algorithm can accurately identify abnormal gaits of groups with different walking habits, and the recognition rate reaches 91.2%. Moreover, the designed PID_SC controller significantly improves the compliance and stability of the robot during assisted walking. Considering the convenience and comfort that the method offers by not requiring patients to wear sensors that introduce troublesome step of detection point precorrection, it can be applied to all wheeled walking aid robots with similar structures, and popularized to help the elderly and the disabled in hospitals, home and other places.

In the next step, we will further explore the safety of wheeled walking aid robot. Our purpose is to develop an environmental and gait information-based controller which will encourage safety in small space areas such as homes.

## Figures and Tables

**Table 1:** Parameters of the membership function.

Input parameter	Low		Middle			High	
Dev	12	16	14	20	26	22	28
*z*	5	16	11	18	24	21	27
*f*	1	3	2	4	6	5	6

**Table 2:** The contrast results of the NIFPN and SVM.

Events	Action times		Detected abnormal gait		Detected nonabnormal		Accuracy rate	
	NIFPN	SVM	NIFPN	SVM	NIFPN	SVM	NIFPN	SVM
Forward falling	30		25	25	0	0	100%	100%
Backward falling	30		29	25	1	0	96.7%	100%
Sideway falling	30		25	27	5	3	83.3%	90%
Vertical falling	30		27	29	3	1	90%	96.7%
Walking	30		0	0	7	1	100%	100%
Drag to gait	30		26	12	4	18	86.7%	40%

**Table 3:** Comparison of gait phase between compliant robotic-assisted walking and passive walking.

Parameter	Affected side		Nonaffected side	
	Compliant assisted	Passive assisted	Compliant assisted	Passive assisted
Support phase	2.11 ± 0.74	1.62 ± 0.52	1.96 ± 0.75	1.57 ± 0.38
Initial leg support	0.46 ± 0.56	0.37 ± 0.37	0.74 ± 0.84^*∗∗*^	0.24 ± 0.36
Single leg support	1.21 ± 0.45^*∗∗*^	0.57 ± 0.65	0.97 ± 0.51	0.86 ± 0.58
End leg support	0.75 ± 0.46^*∗∗*^	0.22 ± 0.46	0.46 ± 0.75	0.34 ± 0.46
Swing phase	0.89 ± 0.37	0.79 ± 0.47	1.31 ± 0.45^*∗∗*^	0.71 ± 0.15

**Table 4:** Comparison of contact area and pressure between compliant robotic-assisted walking and passive walking.

Parameter	Position	Affected side		Nonaffected side	
		Compliant assisted	Passive assisted	Compliant assisted	Passive assisted
Contact area (cm^2^)	Forefoot	29.75 ± 14.48	25.74 ± 17.76	32.46 ± 13.25	29.85 ± 14.65
	Mid-foot	46.74 ± 15.37^*∗∗*^	31.37 ± 16.84	43.64 ± 13.65	40.75 ± 16.75
	Rear foot	30.44 ± 13.52	26.43 ± 12.64	31.65 ± 17.75	33.01 ± 12.36
	Full foot	106.93 ± 26.74^*∗∗*^	83.54 ± 24.16	107.75 ± 23.33	103.61 ± 32.03

Contact pressure (10^3^ kPa)	Forefoot	48.36 ± 39.96	51.25 ± 41.64	87.37 ± 47.76	77.86 ± 81.04
	Mid-foot	94.64 ± 48.64^*∗∗*^	59.96 ± 39.63	92.73 ± 58.76^*∗*^	71.65 ± 74.18
	Rear foot	59.37 ± 36.52	42.65 ± 26.37	89.97 ± 71.27	70.75 ± 75.64
	Full foot	202.37 ± 133.05^*∗*^	153.86 ± 77.27	270.07 ± 85.74^*∗∗*^	220.26 ± 94.72

Peak pressure (10^3^ kPa)		218.53 ± 135.64	172.36 ± 82.46	310.73 ± 128.17^*∗∗*^	259.36 ± 127.84

**Table 5:** Comparison of asymmetric index between compliant robotic-assisted walking and passive walking.

Parameter	Compliant assisted	Passive assisted
Support time	0.12 ± 0.47^*∗*^	−0.46 ± 0.78
Swing time	−0.55 ± 1.19^*∗*^	0.41 ± 0.69
Contact area	−7.54 ± 19.65^*∗*^	−27.76 ± 16.35
Contact pressure	−38.75 ± 53.46	−45.54 ± 35.57

**Table 6:** : Comparison of human robot interaction methods.

Parameter	Pressure sensor	Gyroscope	The proposed mehtod
Wearable devices	Special wearable devices	Special wearable devices	No
Wear position	Insole	Limb or trunk	No
Compliant control	ⓧ	√	√
Abnormal gait	√	√	√
Drag-to gait	ⓧ	ⓧ	√
Accuracy	90%	95.83%	91.2%
Misidentification rate	18.18%	0.89%	6.27%

**Table 7:** Comparison of subjective parameters of different human-robot interaction methods.

Subject	Score (traditional	Trend	Classification
	Noncontact		Results
1	4/6	↑	74.53
2	3/5	↑	81.36
3	5/5	=	63.13
4	3/4	↑	69.64
5	4/6	↑	70.34
6	3/4	↑	75.24
7	1/5	↑	87.37
8	2/6	↑	86.43
9	4/3	↓	59.76
10	4/6	↑	81.35
11	4/6	↑	69.76
12	2/4	↑	73.58
13	4/4	=	55.67
14	2/4	↑	76.53
15	2/6	↑	81.87
16	3/6	↑	72.76
17	2/4	↑	57.34
18	3/4	↑	79.48
19	4/3	↓	82.84
20	5/6	↑	75.78

## Data Availability

The data that support the findings of this study are available from the corresponding author, upon reasonable request.
